# Conservative management of bullet lodged in the interventricular septum of the heart: a case report

**DOI:** 10.1186/s13256-025-05716-y

**Published:** 2025-11-28

**Authors:** Pankaj Aggarwal, Sanketh Edem, Sachin Mahajan, Uday Tej, Jigyasa Bharti

**Affiliations:** Department of Cardiothoracic and Vascular Surgery, Post Graduate Institute of Medical Education and Research, Chandigarh, India

**Keywords:** Gunshot wounds, Cardiac trauma, Interventricular septum, Conservative management, Retained cardiac bullet

## Abstract

**Background:**

Penetrating cardiac injuries from gunshot wounds are rare but potentially fatal. When a bullet is retained within the heart, surgical removal is usually recommended owing to risks of tamponade, arrhythmia, embolization, or infection. However, conservative management may be an option in select hemodynamically stable patients. We present a unique case to highlight this approach.

**Case presentation:**

A 37-year-old South Asian male sustained a gunshot wound to the right hemithorax, resulting in a bullet lodged in the interventricular septum. Diagnostic imaging confirmed the bullet’s stable position with no pericardial effusion or significant cardiac dysfunction. He was managed conservatively with an intercostal chest drain for associated hemothorax, broad-spectrum antibiotics, and close monitoring. A multidisciplinary team including trauma surgeons, cardiologists, and cardiothoracic surgeons determined that nonoperative management was feasible. The patient remained asymptomatic during hospitalization and follow-ups for 1 year, with no evidence of bullet migration, embolism, or infection.

**Conclusion:**

This case report demonstrates that, in carefully selected, hemodynamically stable patients with retained intracardiac bullets, conservative management may be a safe and effective alternative to surgical removal. It underscores the importance of individualized decision-making and close multidisciplinary follow-up.

**Supplementary Information:**

The online version contains supplementary material available at 10.1186/s13256-025-05716-y.

## Introduction

Penetrating thoracic injuries remain a significant source of morbidity and mortality, with gunshot wounds (GSWs) posing a particularly grave threat owing to their potential to injure vital intrathoracic structures. Among these, cardiac involvement is especially critical and often fatal, owing to the heart’s central anatomical location and the potential for rapid hemodynamic collapse. Although most cardiac GSWs necessitate urgent surgical intervention, the management strategy becomes less straightforward when a patient is hemodynamically stable and the projectile is retained within the heart without causing acute complications [1].

Intracardiac foreign bodies such as bullets may lead to a variety of adverse outcomes including cardiac tamponade, arrhythmias, infection, embolism, and myocardial perforation. Accordingly, surgical removal is traditionally advised to prevent delayed complications. However, in select cases, particularly when the foreign body is stable and not impinging on critical cardiac structures, conservative (nonsurgical) management may be feasible [2].

This report presents a rare case of a 37-year-old male with a bullet lodged in the interventricular septum who was managed conservatively with excellent outcomes. The case underscores the importance of individualized decision-making, supported by advanced imaging and multidisciplinary input.

## Case presentation

A 37-year-old South Asian male, with no significant past medical or surgical history and no known comorbidities presented to the Trauma Emergency Department at PGIMER Chandigarh with a gunshot wound sustained to the back of the right hemithorax. The entry wound was located on the mid-back of the right hemithorax. Upon initial evaluation, the patient had stable vital signs with a pulse rate of 82 beats per minute (regular), blood pressure of 130/70 mmHg, saturation of 92% on room air, and an intact airway. However, the patient had reduced air entry in the right lower thorax.

**Fig. 1:**
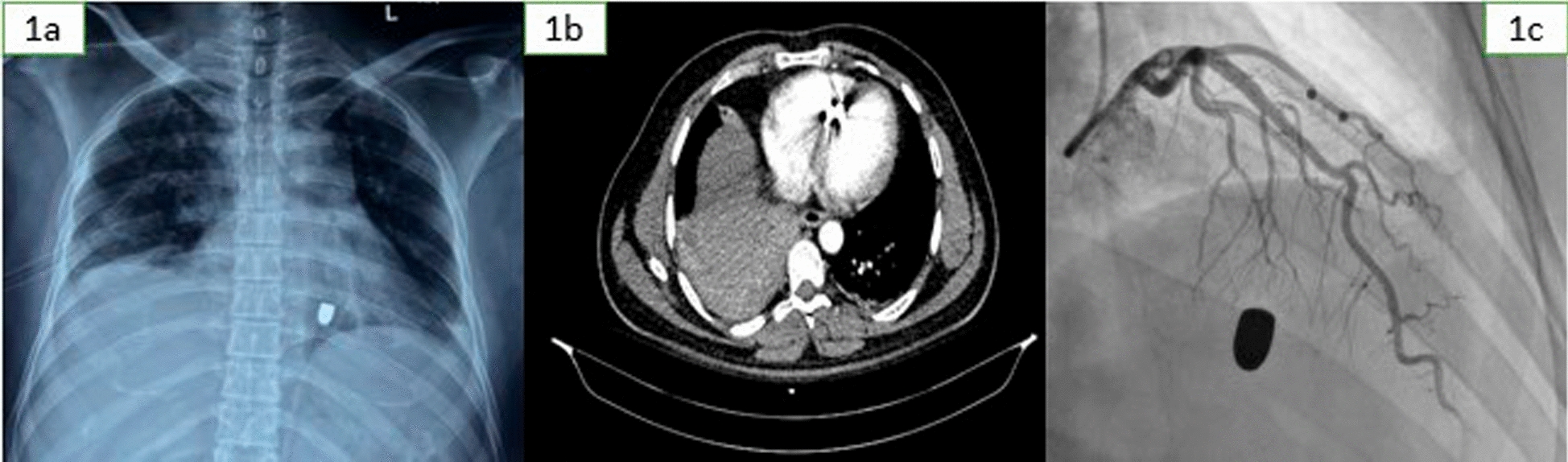
**a** Chest X-ray posteroanterior view showing the bullet in the mediastinum and intercostal chest drain in the right hemithorax. **b** Contrast-enhanced computed tomography of the thorax showing bullet in the interventricular septum. **c** Angiography showing bullet inside the heart with no injury to the coronaries

Imaging played a critical role in confirming the extent of injury and guiding management. A chest X-ray (posteroanterior (PA) view) revealed a radiopaque foreign body in the mediastinum, consistent with a retained bullet. Additionally, a right-sided intercostal chest drain (ICD) was noted in situ. A contrast-enhanced computed tomography (CECT) scan of the thorax and abdomen revealed a hyperdense linear metallic object measuring approximately 18.1 mm embedded in the mid-interventricular septum, consistent with a bullet. Surrounding streak artifacts were noted, but there was no evidence of pericardial effusion. Additional findings included a hyperdense metallic fragment in the posterior basal segment of the right lower lobe of the lung, basal consolidation in the same region, a bicortical fracture of the right ninth rib indicative of the entry point, and a grade 2 hematoma in segment VII of the liver measuring 6.8 × 3.3 cm^2^. A two-dimensional (2D) transthoracic echocardiogram further delineated the cardiac involvement, showing a 15 × 5 mm^2^ hyperechoic structure embedded in the interventricular septum without flow disturbance across the septum or protrusion into cardiac chambers. The study showed no pericardial effusion, preserved left ventricular ejection fraction (55–60%), and trivial mitral and tricuspid regurgitation. No regional wall motion abnormalities were observed. These findings are demonstrated in Fig. 1a–c and Video 1.

## Management

A multidisciplinary team including trauma surgeons, cardiologists, cardiothoracic surgeons, and anesthetists determined the best course of action. The patient was started on broad-spectrum antibiotics and analgesics. An intercostal chest drain (ICD) was placed on the right side to manage the hemothorax, draining approximately 150 mL of blood and alleviating symptoms. The liver injury was managed conservatively.

Holter monitoring revealed no significant arrhythmias. A coronary angiography via a right radial catheter (Tiger 5F) confirmed normal epicardial coronaries and the bullet’s stable position in the mid-interventricular septum (Fig. 1c).

During the hospital stay, the patient remained hemodynamically stable without fever or sepsis. The ICD was removed on day 4. Antibiotics were continued for 7 days before discontinuation.

## Outcome

The patient remained asymptomatic throughout his hospital stay with no complications. A thorough evaluation by the multidisciplinary team confirmed that conservative management was appropriate. The patient was discharged after 7 days in stable condition with scheduled follow-ups.

The patient was evaluated at 7, 14, and 30 days, and subsequently at 3 months and 1 year. Each visit included a clinical examination, electrocardiography (ECG), and transthoracic echocardiography. Imaging consistently demonstrated stable positioning of the retained missile without migration, embolism, pericardial effusion, or ventricular dysfunction. The patient remained asymptomatic throughout follow-up, with no arrhythmias or cardiac compromise. He was monitored for infective endocarditis; no clinical evidence was observed. Although prophylactic antibiotics were not prescribed, counseling was provided regarding the potential symptoms of endocarditis and the need for prompt medical attention should they arise. No further interventions were required.

## Discussion

Penetrating cardiac injuries, particularly those caused by gunshot wounds, are often fatal and demand immediate, decisive intervention. When a foreign body becomes lodged within the heart, the risks of tamponade, embolization, infection, arrhythmia, and structural damage typically prompt surgical removal. However, in hemodynamically stable patients without immediate life-threatening complications, nonoperative or conservative management may be a reasonable alternative, especially when surgical extraction carries its own set of significant risks [3].

In this case, the bullet was embedded within the interventricular septum—a location that poses considerable surgical challenges owing to its proximity to the cardiac conduction system and major coronary vessels. Attempting removal from this region can risk inducing conduction abnormalities, myocardial perforation, or coronary injury [4]. Given the patient’s stable hemodynamic status, absence of pericardial effusion or valvular involvement, and no evidence of bullet migration, conservative management was chosen in consultation with a multidisciplinary team.

Key elements for success include the absence of systemic infection, embolic phenomena, or mechanical interference with intracardiac structures. Regular imaging, cardiac monitoring, and long-term follow-up are essential components of a conservative strategy.

In this patient, comprehensive imaging including CECT, echocardiography, and coronary angiography confirmed the bullet’s stable position and ruled out acute injury to surrounding structures. Serial follow-ups over 1 year confirmed that the bullet had not migrated and no delayed complications occurred. Other reported cases describe patients ranging in age from 15 to 39 years with retained cardiac missiles. The missiles were found in a variety of locations, including free-floating within the left or right ventricular cavities in adolescents and embedded within the interventricular septum in young and middle-aged adults. Patients with intracavitary projectiles generally underwent surgical removal on cardiopulmonary bypass with favorable outcomes, while those with septal-embedded fragments who remained hemodynamically stable were managed conservatively with close surveillance, in one instance supplemented with colchicine for pericarditis prophylaxis. Taken together, these cases highlight that management strategy depends largely on projectile location and clinical stability, with operative intervention favored for mobile intracavitary missiles and nonoperative observation appropriate for stable patients with septal involvement [5–8].

This case adds to the growing body of evidence that, under carefully selected conditions, conservative management of intracardiac missiles can be both safe and effective. It highlights the importance of individualized treatment decisions, multidisciplinary collaboration, and vigilant follow-up.

## Conclusion

This case highlights that conservative management can be a safe and effective option for patients with a bullet lodged in the interventricular septum, provided they are hemodynamically stable and closely monitored. A carefully coordinated multidisciplinary approach enabled us to avoid the risks of surgical intervention without compromising the outcome. While operative removal remains standard in many cases, this report adds to the growing evidence that selected patients can benefit from a nonsurgical approach with excellent long-term results.

## Supplementary Information

Supplementary Material 1. Video 1 2D Echo parasternal long axis view showing bullet in the interventricular septum.

## Data Availability

The data that support the findings of this study are available from the corresponding author upon reasonable request.
